# Under Which Flag?

**Published:** 1891-05-30

**Authors:** 


*For Hospitals, Asylums, Infirmaries, Dispensaries, Medical Practitioners, Students, Nurses, and the Charitable Public.*

Vol. X.—No. 244.] [May 30, 1891.

### Under Which Flag?

There are two rules which govern the conduct of the world. Stated in briefest form the one is "Do as you would be done by"; the other is "Do as you like. A certain portion of the old world used to believe in God and the devil. For all practical purposes, many have given up the devil long ago; and not a few are striving to do without a belief in God. We need not here consider the peculiar forms under which ancient convictions and ancient conduct ranged themselves. The Hospital, as its readers know, stands by the old landmarks; believes honestly and squarely in God, in a Divine revelation, and in Christianity, and thinks it has scientific warrant for so doing. But we do not wish to approach the consideration of Hospital Sunday duty from any standpoint which will detach from us the sympathy of a single well-meaning person of any religious faith, or of no faith at all. The general principle of "doing as you would be done by" commands the approval and more or less of the practical adherence of men of every conceivable creed or intellectual standpoint; the opposite and antagonistic principle of "doing as you like" meets with the intellectual approval of very few, though with the practical adherence of, perhaps, the great majority of mankind.

It is a good thing, and a necessary thing, to get men to fight under one flag or another. Wholeheartedness, strenuousness, are the indispensable conditions of getting the best out of a man that is in him. Dilettantism is of the devil, there is no doubt about that; every man of forty knows it by his own experience. But if the soul and essence of high human conduct be conflict, then that conflict should be waged with the utmost degree of valour and with every resource of skill. This at any rate is what a native born Briton feels, and it is what every man of every race ought to feel who lives in daily association with men and women of British blood, and under the noble protection of Britain's laws and flag.

On Hospital Sunday men and women are not asked to declare themselves Churchmen or Nonconformists, Jews or Christians, Conservatives or Liberals, Englishmen or foreigners. But they are asked to range themselves under the flag whose motto is, "Do as you would be done by"; or under that which bears the other motto, "Do as you like." These two opposed principles, it must be remembered, are trees: each of them has its root, its stem, its branches, its leaves, and its fruit. No man who is worthy of the name of honest thinker can for a moment doubt whether of the two is the wholesomer tree. The fruit of both has qualities that are intellectual, moral, and in every way practical. "Do as you would be done by" is the tree that bears the fruit of discipline, of reasonableness, of intellectual culture, of scientific progress, of practical good behaviour, of peace and pleasure at home and abroad. "Do as you like" bears the fruit of spoilt childhood, of uncultured, selfish and brutal manhood, of greed, of robbery, of murder, of hatred in the family and in the world. There is no doubt whatever about the flag under which every well-conditioned man and woman in the world will choose to be ranged.

Now, if "do as you would be done by" indisputably includes any one obligation, it is the obligation of caring for the helpless sick. When a man is really ill, no wholesome-hearted person can deny him pity; when he is not only ill but poor and helpless, nobody, who is not a brute, can resist the desire to give him help. We have come then to this, that every one of us who is worthy of the name of man or woman must range ourselves under the flag of "Do as you would be done by," and must make up our minds to fight with the utmost possible valour and skill in the special battle which goes by the name of Hospital Sunday. We must be led to the charge, every regiment of us, and every soldier in it. But what shall we do if we have had no previous drill? and if we have not taken the trouble to provide ourselves with even the private soldier's little "Book of Instructions"?

When we look at the subject in this definite way, some of us cannot help feeling that we are very shuffling and shiftless soldiers; and that, however proud we may be of our flag, our flag has very little reason to be proud of us. Even some of us who may be clergy, and who take the command of companies and of regiments, have very little enthusiasm for the battle; not half so much as we have for any possible prospect of promotion. This is bad; bad from the point of view of true English blood; bad from the point of view of loyal Christian faith; bad from the point of view of devotion to the universal flag of "Do as you would be done by." It is bad; let us not flatter ourselves that is anything but bad; rather let us have the grace to be ashamed of it.

But, and this may surprise a good many readers, there are a few leaders in the Hospital Sunday fight who are worthy of the name of soldiers. They prepare themselves for the battle, and they train their troops. Conspicuous among these are the Reverend Canon Fleming and the Reverend Dr. Forrest. They have won the chief laurels in this great philanthropic battle for several years past. "It is because they have rich and benevolent congregations," say the envious and the idle. "It is because they are valiant and strenuous warriors, and prepare themselves for the conflict beforehand," says the stern and admonitory voice of truth. Canon Fleming and Dr. Forrest, it is true, live and work in wealthy neighbourhoods, and have rich men and women in their congregations. But they bring ten times as much to the treasury of the Hospital Sunday Fund as many clergymen who work in neighbourhoods as wealthy as theirs, and whose congregations are as rich. If every clergyman and minister in London would work like these two, the £100,000 needed by the hospitals would be raised at once.

If there be any clergyman or minister who wishes to know why the produce of his own field is so poor compared with that of Canon Fleming, the answer is very plain: it is because he does not plough with Canon Fleming's heifer. This reverend Canon—and Dr. Forrest adopts the same tactics—makes a direct appeal on behalf of the Hospital Sunday Fund to every member of his congregation who can by any possibility be supposed to have anything to spare. He is like Moltke and other great commanders—he lays all his plans carefully many weeks beforehand. Hospital Sunday is but the day when his forces join issue with the enemy: it is also the day when they carry all before them. We have before explained what it is that Canon Fleming does: he prepares with his own hand a special circular letter for each year, and five or six weeks before the day of battle he rallies his forces by sending this letter, over his own signature, to every member of his congregation. Not only do those who are at home receive the letter, but those who are "travelling by land or by water." Everyone is asked, either to be in his place on Hospital Sunday, and to take his personal share in the engagement, or to send his cheque at the right moment, so that it may contribute its quota to the final triumph. This is the way to fight, and it is the way to win. Is the Hospital Sunday collection worth the making? Is this great annual battle of philanthropy worth the fighting? Is this £100,000 a necessary sum to be raised?—these are the questions to ask. If the annual battle be worth the fighting—and what man of any conscience or character will say that it is not?—then it is worth fighting with every resource of preparedness, of courage, of skill, and of generous enthusiasm. He who fights any noble battle whatsoever in another spirit than this is a miserable creature, not worthy to be set "even with the dogs of the flock."

The laity, on Hospital Sunday, occupy the position of the rank and file, the clergy being the captains and generals. The soldiers of this country have always had as keen a relish for fighting as their officers, and as eager an ambition for victory. We English, when put upon our mettle, hate defeat; and we hate indecisive success almost as much. It is unnatural for us to do things by halves, or to do them feebly. A languid Hospital Sunday, a languid parson, languid people, and an impotent collection, are things which we should look upon with contempt. If we have adjured every clergyman to lead and inspire his people bravely and with enthusiasm, still more do we call upon all the people, without whom the clergyman cannot fight at all, to rouse up in themselves their nobler feelings and sympathies, and to resolve, with conscious and intelligent humanity, to make one great and irresistible effort to carry the Hospital Sunday battle to the conquest of a hundred thousand pounds. The thing is eminently worth the doing, fellow citizens of this great London! Let us rise up and do it!

